# Capillary zone electrophoresis as a quality assessment tool of paracetamol and phenylephrine hydrochloride in presence of paracetamol impurities

**DOI:** 10.3906/kim-2107-21

**Published:** 2021-10-11

**Authors:** Joliana F. FARID, Nadia M. MOSTAFA, Yasmin M. FAYEZ, Hebatallah M. ESSAM

**Affiliations:** Analytical Chemistry Department, Faculty of Pharmacy, Cairo University, Cairo, Egypt

**Keywords:** Capillary electrophoresis, Paracetamol, Phenylephrine HCl, p-Aminophenol, p-Nitrophenol

## Abstract

The combination of paracetamol (PAR) and phenylephrine HCl (PHE) is a common pharmaceutical combination intended to manage symptoms of every day cold. Since paracetamol is susceptible to degradation or some manufacturing-related impurities, its stability should be monitored continuously. The developed method in this study for the determination of PAR and PHE in presence of PAR impurities is considered a quality assessment tool especially when PAR impurities can be quantitatively determined. A capillary zone electrophoresis (CZE) method was optimized and validated for simultaneous determination of PAR and PHE in presence of PAR impurities namely, p-Aminophenol (PAP), p-Nitrophenol (PNP), Acetanilide (ACT) and p-Chloroacetanilide (CAC) with further quantification of these toxic impurities. Factors that affect the separation quality such as pH, buffer and applied voltage were optimized. The separation was carried out using 20 mM phosphate buffer (pH 8). The linearity was reached over concentration ranges of 30–250 μg/mL, 1–40 μg/mL, 2–50 μg/mL, 2–50 μg/mL, 2–50 μg/mL and 2–50 μg/mL for PAR, PHE, PAP, PNP, ACT, and CAC, respectively, with accuracy ranging from 99.06% to 100.62%. After validation, the method was applied for pharmaceutical formulation analysis with RSD <2%. Moreover, statistical comparison with the official methods confirms that the method is a viable alternative for quality assessment of this combination.

## 1. Introduction

Many pharmaceutical formulations are used to treat common cold symptoms. Paracetamol (PAR) and phenylephrine HCl(PHE) combination is one of these formulations intended to control symptoms.

**Figure 1:**
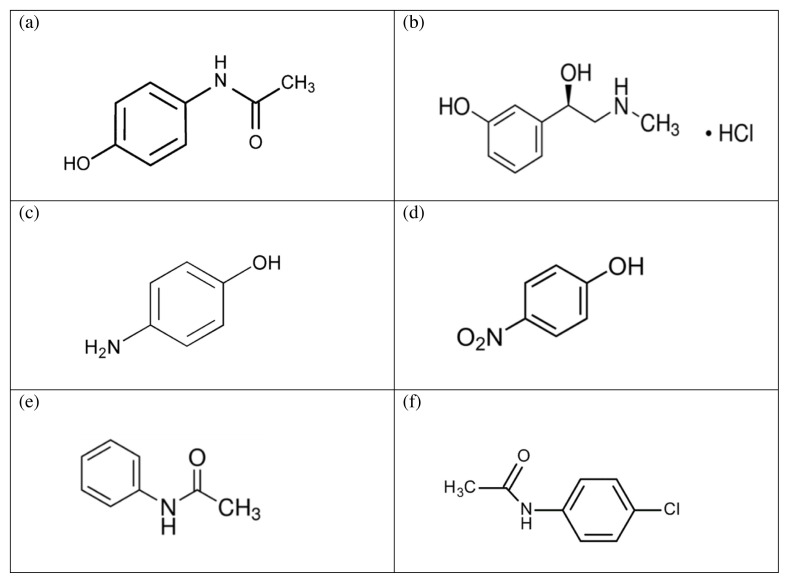
Chemical structure of (a) Paracetamol, (b) Phenylephrine HCl, (c) p-Aminophenol, (d) p-Nitrophenol, (e) Acetanilide, (f) p-Chloroacetanilide.

Paracetamol (PAR), N-(4-hydroxyphenyl) acetamide (Figure 1a) is a popular analgesic and antipyretic [ 1]. It is an essential nonsteroidal antiinflammatory medicine that is used as pain killer and fever treatment in adults and children formulations. It is an official drug in British [1] and United States [2] pharmacopoeias.

Paracetamol can be used alone or with other constituents, e.g., antihistamines and/or decongestants. Phenylephrine HCl (PHE), (1R)-1-(3-hydroxyphenyl)-2-(methylamino) ethanol hydrochloride [1] (Figure 1b) has α-adrenergic activity [3]. Thus, it is used to relieve congestion. It is an official drug in British [1] and United States [2] pharmacopoeias.

Paracetamol is susceptible to degradation or may have some impurities that are process-related and appear during the manufacture. P-aminophenol (PAP), (Figure 1c) is PAR primary impurity with a nephrotoxic effect [4, 5 ]. It has a teratogenic ability [ 6, 7 ]. According to British Pharmacopoeia, it is considered to be an impurity “K” for PAR and must not exceed 50 ppm for PAR drug substance and 0.1% (1000 ppm) for PAR tablets [ 1], so its quantity must be controlled. P-nitrophenol (PNP), (Figure 1d) is a toxic impurity like many nitrophenols [8] and can cause methemoglobinemia whichis characterized by impairment of carrying oxygen to tissues [6]. It was mentioned in the British Pharmacopoeia (B.P.) tobe an impurity “F” for PAR with a limit of 0.05% (500 ppm) for PAR drug substance and 0.25% (2500 ppm) for PAR tablets [1].

Furthermore, acetanilide (ACT) (Figure 1e) is another PAR impurity that is considered a PAR impurity “D” with a limit of 0.05% (500 ppm) according to British Pharmacopoeia [1]. Anemic anoxia is one of its characteristic toxic symptoms [9]. P-chloroacetanilide (CAC) (Figure 1f) is known as PAR impurity “J” and must not exceed 10 ppm as mentioned in BritishPharmacopoeia [1]. It may adversely affect skin and eye that may lead to skin and eye damage [5, 7 ]. Also, it may causehemolysis with hepatotoxicity and nephrotoxicity [ 7, 10 ].

These impurities which may be involved during synthesis or produced as degradation products should be monitored. Thus, the development of a method of analysis for determination of the active ingredients in presence of impurities is needed with further quantification of these toxic impurities.

To our knowledge, few methods were reported for determination of PAR and PHE simultaneously including chromatographic [ 11, 12 ], spectrophotometric [ 13–15], and electrochemical methods [16]. However, the literature lacks the presence of any capillary electrophoresis method for the simultaneous determination of the two mentioned drugs.

The present work is the first capillary zone electrophoresis (CZE) method that developed for separation of PAR, PHE, and PAR impurities: PAP, PNP, ACT, and CAC and is considered a quality assessment tool for further quantification of these toxic impurities.

## 2. Experimental

### 2.1. Apparatus

CZE was performed using an Agilent G 7100A-DAD equipped with an automatic injector and an autosampler, coupled with a photodiode array detector. A bare fused-silica capillary (Agilent Technologies, Germany) 50 μm id, with effective length of 25 cm was used. Sonicator (Memmert, Germany). Jenway 3505 pH-meter (Jenway, UK), was employed for pH adjustment.

### 2.2. Standards

- Paracetamol was supplied by Amoun Pharmaceutical Co., Egypt.
- Phenylephrine HCl was obtained from Sigma Pharmaceutical Industries, Egypt.
- P-Aminophenol, certified to contain 99.5%, was purchased from Chemajet, Alexandria, Egypt.
- P-Nitrophenol, certified to contain 99%, was purchased from Sigma Aldrich, Darmstadt, Germany.
- Acetanilide, certified to contain 99%, was purchased from Universal fine chemicals, Raheja, Mumbai.
- P-Chloroacetanilide, certified to contain 99%, was purchased from Universal fine chemicals, Raheja, Mumbai.
- Tylenol^®^ sinus tablets (Batch No. C001K6) labeled to contain 500 mg PAR and 5 mg PHE (Johnson & Johnson INC., Canada).

### 2.3. Chemicals and reagents

- Analytical grade chemicals and HPLC grade solvents were used.
- Sodium hydroxide pellets, phosphoric acid, dipotassium hydrogen phosphate and methanol were purchased from Sigma-Aldrich, Germany.
- Deionized water is from Egypt Otsuka Pharmaceutical Co. Cairo, Egypt.

### 2.4. Solutions

- Stock solution of PAR (5 mg/mL) was prepared in methanol.
- Stock solutions of PHE, PAP, PNP, ACT and CAC (1 mg/mL) were prepared in methanol.
- Working solution of PAR (1 mg/mL) was prepared from corresponding stock solution in 20 mM phosphate buffer (pH 8) as background electrolyte (BGE).
- Working solutions of PHE, PAP, PNP, ACT and CAC (100 μg/mL) were prepared from their corresponding stock solutions in 20 mM phosphate buffer (pH 8) as background electrolyte (BGE).

### 2.5. Procedures

#### 2.5.1. Construction of calibration curves

Suitable aliquots were accurately transferred from PAR, PHE, PAP, PNP, ACT and CAC working solutions into a set of 10-mL volumetric flasks and the volumes were completed to the mark with 20 mM phosphate buffer (pH 8) to construct calibrations for PAR, PHE, PAP, PNP, ACT, and CAC in the range of 30–250 μg/mL, 1–40 μg/mL, 2–50 μg/mL, 2–50 μg/mL, 2–50 μg/mL, and 2–50 μg/mL, respectively. Calibration curves were the relation between peak areas and the concentrations of each component and the regression equations were calculated.

#### 2.5.2. Capillary electrophoretic procedure

A bare fused-silica capillary with 50 μm id, and effective length of 25 cm was used. Each run started with preconditioning, then injection of the sample was done and lastly postconditioning was conducted. Preconditioning was implemented by flushing with 0.1 M NaOH, water and BGE for 5 min, respectively. Samples were filtered through a 0.22 μm millipore membrane filter then introduced into the capillary hydrodynamically using a pressure of 50 mbar for 5 s with UV detection at 200 nm and separation voltage of 20 kV and a stable temperature of 25 °C. As postconditioning, water was flushed for 5 min.

#### 2.5.3. Application to pharmaceutical formulation

The film coat was carefully removed using methanol and the contents of ten tablets were accurately weighed and powdered. A weight equivalent to one tablet was transferred into a 100-mL volumetric flask, complete to the mark with methanol and sonicated for 15 min. The solution was filtered and aliquots were transferred to 50 mL volumetric flasks. The volume was completed. The general procedure previously mentioned was applied to quantify each drug concentration in the dosage form solutions.

For applying the standard addition technique, known amounts of each standard component were separately added to aliquots of the prepared tablet solution and concentration of the added standard was determined after subtraction.

To confirm the validity of the proposed CZE method, the results obtained by applying the developed method were statistically compared with the official methods. PAR is determined according to the united states of pharmacopoeia [2] by direct UV spectrophotometric at 244 nm, while PHE determination depends on a potentiometric titration method using sodium hydroxide as a titrant according to the British pharmacopoeia [1].

## 3. Results and discussion

Pharmaceutical compounds that are susceptible to degradation or may have impurities produced during synthesis should be monitored to control the percentage of degradation products or impurities especially when they are toxic. PAR is prone to degradation and may have impurities like PAP, PNP, ACT, and CAC. Those impurities are toxic and can adversely affect human health. Thus, they should be monitored and quantified along with PAR and PHE. The literature reveals that few methods were developed for PAR and PHE determination. Only chromatographic [11, 12 ], spectrophotometric [13–15], and electrochemical [16] methods were previously reported for PAR and PHE determination in pharmaceutical formulations. Therefore, it was very important to develop accurate methods that can be used for determination of the active compounds and used as quality assessment tool for quantification of the impurities that may present in the pharmaceutical formulations.

### 3.1. Method development and optimization

Many factors affect the electrophoretic separation but pH, buffer type, and applied voltage are the main factors that should be optimized. Hence, all the experimental conditions and parameters that can affect the electrophoretic separation were investigated and optimized to reach optimum separation between the studied drugs and impurities.

Firstly, borate and phosphate buffers were investigated at pH of 5.5, 6.5, and 8. Borate buffer produced bad resolution with slow migration rates. On the other hand, good separation was achieved upon using phosphate buffer. Buffers with pH 5.5 and 6.5 showed poor peak shape and poor resolution, while buffer with pH 8 was the optimum in producing well-resolved peaks with good accuracy and precisions of the components to be separated in a short time. According to the ionization and charge/size of the components, the expected order of elution is PHE, PAP, ACT, PAR, CAC, and PNP.

The BGE concentration effect on separation was tested in the range from 20 to 50 mM. The migration time was delayed when the concentration of the buffer increased. Also, an increase in current was observed while increasing buffer molarity that may cause joule heating leading to nonuniform temperature gradient, local changes in viscosity and subsequent zone broadening. Finally, 20 mM phosphate buffer (pH 8) gave sharp peaks with optimum resolution.

**Figure 2:**
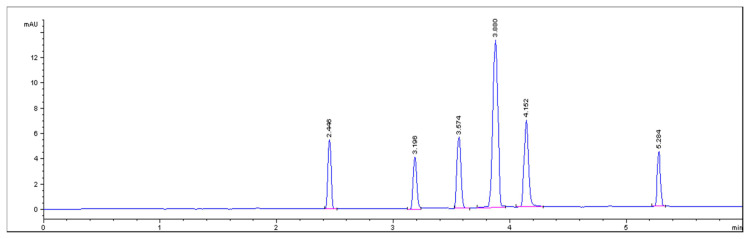
Capillary zone electrophoresis (CZE) electropherogram of PHE (MT=2.446), PAP (MT=3.196), ACT (MT=3.574), PAR (MT=3.880), CAC (MT=4.152), and PNP (5.284) using 20 mM phosphate buffer pH 8 as BGE and 20 kV at 200 nm.

Voltages of 15–25 kV were tried. Generally, higher voltages mean more efficient separation with better resolution and shorter migration times. Although increasing the voltage results higher current, while decreasing the voltage less than 20 kV produced less defined peaks. 20 kV permits efficient separation with reasonable current intensity. Detection was tried at three different wavelengths 200, 210, and 257 nm. The best sensitivity was obtained at 200 nm (Figure 2).

### *3.2*. Method validation

The proposed method was validated according to ICH guidelines (Table 1). The linearity of the studied components was assessed between the peak area and the related concentrations of PAR, PHE, PAP, PNP, ACT, and CAC. Linear regressions are obtained in the range of 30–250 μg/mL, 1–40 μg/mL, 2–50 μg/mL, 2–50 μg/mL, 2–50 μg/mL and 2–50 μg/mL for PAR, PHE, PAP, PNP, ACT and CAC, respectively.

Accuracy was determined using synthetic mixtures containing all components under investigation in different ratios at three concentration levels for each in three replicates. The accuracy mean percentage recoveries of PAR, PHE, PAP, PNP, ACT, and CAC were 100.34 ± 0.69, 100.62 ± 0.50, 99.06 ± 0.45, 99.53 ± 0.66, 100.48 ± 0.32, and 99.24 ± 0.83, respectively. The obtained results proved that the developed methods are accurate and reliable.

Precision was also tested with respect to the intraday (repeatability) and interday (intermediate) variations. Intraday precision was tested in triplicate within short interval times in the same day while intermediate precision was determined on three successive days. The obtained RSD values were <2%, indicating good precision.

LOD and LOQ were evaluated using the mathematical method. LOD was calculated using 3.3×SD/slope, the LOD obtained were 6.44 μg/mL, 0.17 μg/mL, 0.54 μg/mL, 0.57 μg/mL, 0.64 μg/mL, and 0.39 μg/mL for PAR, PHE, PAP, PNP, ACT, and CAC, respectively. Whereas LOQ is ten times the standard deviation of response divided by the slope, they were 19.51 μg/mL, 0.52 μg/mL, 1.65 μg/mL, 1.74 μg/mL, 1.93 μg/mL, and 1.18 μg/mL for PAR, PHE, PAP, PNP, ACT, and CAC, respectively.

Robustness of the analytical method evaluates the capability of the method to retain its validity under deliberate experimental variation. Factors explored in robustness analysis are acidity of the background electrolyte and the applied voltage. Results of recovery % and RSD % demonstrate sufficient robustness as in Table 1.

An overall system suitability testing was calculated (Table 2). Satisfactory results regarding the system suitability parameters including resolution (RS), tailing factor (T), selectivity factor (α) and column efficiency (N) were obtained for the proposed CZE method.

### 3.3. Application to pharmaceutical formulation

**Figure 3:**
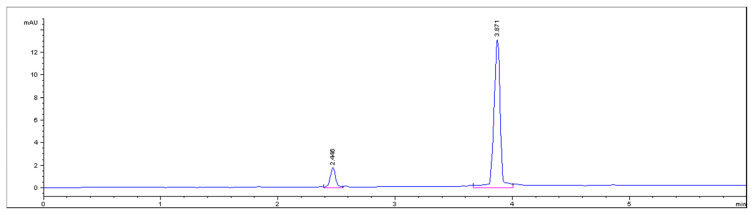
Capillary zone electrophoresis (CZE) electropherogram of the pharmaceutical formulation of PHE (MT=2.440), PAR (MT=3.871) using 20 mM phosphate buffer pH 8 as BGE and 20 kV at 200 nm.

The proposed CZE method has been successfully employed for the simultaneous determination of PAR and PHE in their pharmaceutical formulation (Figure 3). The obtained percentage recoveries of PAR and PHE were 98.17 ± 0.794 and 100.19 ± 0.803, respectively (Table 3).

The standard addition technique was applied (Table 3). Good results validate the fact that the excipients (cellulose, corn starch, magnesium stearate, sodium starch glycolate) did not interfere in the determination of the active ingredients. Finally, the results obtained by applying the proposed CZE method for the determination of PAR and PHE were statistically compared with the official methods [1, 2] and showed no significant difference with respect to accuracy and precision (Table 4).

Capillary electrophoresis has the advantages of reduced sample, costs, and analysis time. The ability to separate a wide range of solutes using a single set of operating conditions is a strong advantage of capillary electrophoresis. This work demonstrates the use of CZE for main peak assay and the validated performance of the results achieved. The specific factors relating to optimized accuracy and precision in CZE assay are discussed. The optimization and selection of suitable conditions to get satisfactory precision, accuracy and linearity is experimentally proven by results from the analysis of different pharmaceutical samples.

## 4. Conclusion

The effectiveness of capillary zone electrophoresis for the separation of PAR and PHE in their binary mixtures, pharmaceutical dosage form and in the presence of PAR related impurities PAP, PNP, ACT, and CAC with further quantification of these toxic impurities has been demonstrated. This simple electrophoretic method is the first method developed for simultaneous determination of this combination in the presence of PAR related impurities and hence, it can be considered a quality assessment tool. The method offers high sensitivity enough to separate PAR from its related structure impurities and coformulated drug in short analysis time with no significant losses in resolution. Good resolution was obtained between the proposed components. The presented CZE method is reproducible and accurate. The proposed method was found to be valid with respect to precision, accuracy, and system suitability. Furthermore, the CZE method is characterized by broad applicability, use of minimal solvents volume, and short analysis time with no need of data manipulation. From the promising results obtained, it can be expected that there will be great potential in the use of the developed method for stability indicating analysis of other paracetamol containing pharmaceutical formulations in quality control laboratories.

## Figures and Tables

**Table 1:** Assay and validation parameters for the determination of PAR, PHE, PAP, PNP, ACT, and CAC by the proposed CZE method.

Parameter	PAR	PHE	PAP	PNP	ACT	CAC
Range (μg/mL)	30–250	1–40	2–50	2–50	2–50	2–50
Slope	0.9564	2.6109	2.0452	1.2255	3.8473	7.1501
Intercept	11.721	1.9132	–0.2564	0.6701	0.6671	–1.1102
Mean of recovery of calibration	99.86	100.05	100.17	99.97	99.78	100.27
SD of recovery of calibration	0.660	0.649	1.071	0.870	0.626	0.708
Correlation coefficient (r)	0.9999	1.0000	0.9999	0.9999	0.9999	1.0000
Accuracy	100.34 ± 0.69	100.62 ± 0.50	99.06 ± 0.45	99.53 ± 0.66	100.48 ± 0.32	99.24 ± 0.83
Repeatability*	99.47 ± 0.63	99.44 ± 0.89	99.32 ± 0.58	99.80 ± 0.73	100.72 ± 0.43	99.49 ± 0.67
Intermediate precision*	99.34 ± 0.82	99.59 ± 1.21	100.22 ± 0.89	100.08 ± 1.08	98.90 ± 0.80	100.29 ± 1.10
LOD (μg/mL)	6.44	0.17	0.54	0.57	0.64	0.39
LOQ (μg/mL)	19.51	0.52	1.65	1.74	1.93	1.18
Robustness**	99.04 ± 0.95	99.89 ± 1.13	98.72 ± 1.66	100.64 ± 1.31	99.88 ± 0.65	100.41 ± 0.84

* Mean values ± RSD of three concentrations of PAR, PHE, PAP, PNP, ACT and CAC analyzed intradaily in triplicate (repeatability) and on three successive days (intermediate precision)

** Factors explored: acidity of the BGE (pH 8.0 ± 0.1) and applied voltage (20 ± 1 kV).

**Table 2:** Parameters required for system suitability tests of CZE method.

	PHE	PAP	ACT	PAR	CAC	PNP	Reference values [17,18]
Migration time (min)	2.446	3.196	3.574	3.880	4.152	5.284	---------
Resolution (R_s_)	11.538		5.617	3.908	3.335	16.526	R_s_< 1.5
Tailing factor (T)	1.10	1.15	1.13	0.98	1.03	1.08	T = 0.8–1.2
Selectivity factor (α)	1.31		1.12	1.09	1.07	1.27	α < 1
Column efficiency (N)	25836.43	38424.36	35302.76	31855.11	47644.66	105031.30	Increase with efficiency of the separation

**Table 3:** Determination of PAR and PHE in its dosage form and application of standard addition technique using the proposed CZE method.

	PAR*	PHE*
Tylenol^®^ sinus **	98.17 ± 0.79	100.19 ± 0.80
Recovery of standard added %**	101.03 ± 0.86	100.07 ± 0.92

* Found % ± SD.

** Average of three determinations.

**Table 4:** Statistical comparison for the results obtained by the proposed method and the official methods for the determination of PAR and PHE in pure powder form.

	CZE		Official method	
	PAR	PHE	PAR*^[2]^	PHE**^[1]^
Mean	99.86	100.05	100.16	100.18
S.D.	0.660	0.649	0.792	0.952
Variance	0.436	0.421	0.627	0.906
n	7	7	6	6
Student’s t-test*** (2.201)	0.728	0.283		
F value***(4.39)	1.44	2.152		

* Direct UV spectrophotometric determination at 244 nm.

** Potentiometric titration method.

*** Figures between parentheses represent the corresponding tabulated values of t and F at p = 0.05.
